# Inhaled Corticosteroid Use and Risk of *Haemophilus influenzae* Isolation in Patients with Bronchiectasis: A Retrospective Cohort Study

**DOI:** 10.3390/jcm14238557

**Published:** 2025-12-02

**Authors:** Dil Afrose, Christian Philip Rønn, Josefin Eklöf, Anna Kubel Vognsen, Louise Lindhardt Tønnesen, Barbara Bonnesen Bertelsen, Jonas Bredtoft Boel, Christian Østergaard Andersen, Ram Benny Christian Dessau, Mette Pinholt, Jens-Ulrik Jensen, Pradeesh Sivapalan

**Affiliations:** 1 Copenhagen Respiratory Research (COP: RESP), Department of Medicine, Herlev and Gentofte Hospital, University of Copenhagen, 2900 Hellerup, Denmark; afrose.dil@gmail.com (D.A.); christian.roenn@regionh.dk (C.P.R.); josefin.viktoria.ekloef@regionh.dk (J.E.); anna.kubel.vognsen.01@regionh.dk (A.K.V.); louise.lindhardt.toennesen@regionh.dk (L.L.T.); barbara.bertelsen@regionh.dk (B.B.B.); jens.ulrik.jensen@regionh.dk (J.-U.J.); 2 Department of Clinical Microbiology, Herlev and Gentofte Hospital, University of Copenhagen, 2730 Herlev, Denmark; jonas.bredtoft.boel.01@regionh.dk; 3 Department of Diagnostic and Infectious Disease Preparedness, Statens Serum Institut, 2300 Copenhagen, Denmark; ccca@ssi.dk; 4 Research Unit for Clinical Microbiology, Zealand University Hospital, 4200 Slagelse, Denmark; ramd@regionsjaelland.dk; 5 Institute of Regional Health Research, University of Southern Denmark, 5230 Odense, Denmark; 6 Department of Clinical Microbiology, Hvidovre University Hospital, 2650 Hvidovre, Denmark; mette.pinholt@regionh.dk; 7 Department of Clinical Medicine, Faculty of Health and Medical Sciences, University of Copenhagen, 2200 Copenhagen, Denmark

**Keywords:** non-cystic fibrosis bronchiectasis, bronchiectasis, inhaled corticosteroids, *Haemophilus influenzae*, *H. influenzae*, ICS use

## Abstract

**Background:** Non-cystic fibrosis bronchiectasis (BE) is a chronic lung condition characterized by irreversible bronchial dilation and presented with persistent respiratory symptoms, recurrent respiratory infections, and decreased quality of life. Inhaled corticosteroids (ICSs) are frequently prescribed in patients with bronchiectasis, despite limited evidence supporting their clinical efficacy. Inhaled corticosteroids have been associated with increased risk of respiratory infection with *Haemophilus influenzae (H. influenzae*) in other groups of lung diseases. We aimed to evaluate the association between ICS use and the risk of isolating *H. influenzae* from lower respiratory tract samples in patients with bronchiectasis. **Methods:** A retrospective cohort study was conducted using data from 2010 to 2018, encompassing all patients diagnosed with bronchiectasis in outpatient clinics in Eastern Denmark. ICS use was standardized in budesonide equivalent doses and categorized in tertiles: low (<210 μg/day), moderate (211–625 μg/day), and high (≥626 μg/day) based on cumulative budesonide equivalent doses redeemed in the 12 months before cohort entry. The primary outcome was the first isolation of *H. influenzae* from lower respiratory tract samples post-cohort entry. Cox proportional hazards models, adjusted for relevant confounders, estimated hazard ratios (HRs), and inverse probability-of-treatment weighting (IPTW) was used in sensitivity analyses. **Results**: Among 3663 patients (mean age 66 years; 61% female), 2175 (59.4%) did not use ICS, while 484 (13.2%), 508 (13.9%), and 496 (13.5%) were in the low-, moderate-, and high-dose ICS groups, respectively. Furthermore, 594 (16.22%) patients had a lower respiratory tract culture positive for *H. influenzae* during follow-up. High-dose ICS use was associated with an increased risk of *H. influenzae*; HR 1.63 (95% Cl, 1.19 to 2.12, *p* < 0.005) compared with no ICS use. No association for low or moderate ICS use was found: low-dose ICS HR 0.75 (95% Cl, 0.52 to 1.07, *p* = 0.11) and moderate-dose ICS HR 1.27 (95% Cl, 0.93 to 1.72, *p* = 0.12). IPTW analysis confirmed the main finding. **Conclusions:** High-dose ICS use in patients with bronchiectasis was associated with an increased risk of acquiring *H. influenzae* in the lower respiratory tract. Hence, patients with bronchiectasis should be cautiously prescribed high-dose ICS.

## 1. Introduction

Non-cystic fibrosis bronchiectasis (BE) is a chronic respiratory disease characterized radiologically by abnormal and permanent widening of the bronchi, accompanied by related clinical symptoms such as a persistent productive cough and recurrent respiratory infections [1,2,3]. BE, formerly known as an orphan disease [4], is now recognized as a common and increasingly prevalent condition worldwide, with reported prevalence rates up to 680 per 100,000 persons [5,6]. The etiology remains unknown in 20–40% of patients [7]. BE frequently coexists with other chronic respiratory conditions, particularly asthma [8] and chronic obstructive pulmonary disease (COPD) [9], which may amplify airway inflammation and worsen prognosis. The pathophysiology of BE is often described by the “vicious vortex” model, in which persistent infection, airway inflammation, impaired mucociliary clearance, and structural damage perpetuate one another [10]. This cycle contributes to declining lung function, increased morbidity and mortality, and reduced quality of life [11]. Consequently, BE imposes a considerable clinical and socioeconomic burden on both patients and the healthcare system worldwide [12]. Current treatment strategies focus on symptom control, reducing exacerbation frequency, and preventing further lung damage [13]. Given these challenges, understanding modifiable risk factors for airway colonization is of major clinical relevance.

Inhaled corticosteroids (ICSs) frequently used in the treatment of BE reduce symptoms and exacerbation frequency through their anti-inflammatory properties, though the mechanism is poorly understood [14,15]. European Respiratory Society guidelines for the management of BE do not recommend prescribing ICSs to patients with BE due to limited supporting evidence and concerns about potential harm [14,16]. ICSs are only indicated in BE when asthma and/or COPD is also present [16]. The known adverse effects of ICSs include infections such as oropharyngeal candidiasis, dysphonia, as well as increased risk of pneumonia [11,17]. Corticosteroids, whether local or systemic, suppress the inflammatory response to infection and inhibit the intracellular infection [18], thereby increasing susceptibility to respiratory infections such as pneumonia [19]. *Haemophilus influenzae* (*H. influenzae*) is a Gram-negative coccobacillus and a common human commensal that colonizes the upper and lower airways [19,20]. It has the ability to colonize the airways of patients with chronic pulmonary suppurative diseases [20]. Chronic colonization with *H. influenzae* contributes to airway inflammation and frequent, predominantly outpatient-managed exacerbations, adding to morbidity [21,22].

We hypothesized that higher ICS doses would be associated with increased risk of lower respiratory tract *H. influenzae* isolation in patients with bronchiectasis. Therefore, this study aimed to evaluate the association between ICS use and risk of lower respiratory tract isolation of *H. influenzae* in patients with BE, with a particular focus on potential dose–response relationships, using a large retrospective cohort from Eastern Denmark during 2010–2018.

## 2. Materials and Methods

### 2.1. Data Sources

Data from regional and nationwide administrative registries were accessed. Unique personal identification numbers were used to link participants across registries, thus ensuring exact linkage on the patient level and allowing complete follow-up until the primary outcome was obtained or the end of the study period. The following registers were used:

1. The Danish National Patient Registry (DNPR), which includes all hospital admissions since 1977 and outpatient data since 1995, was applied to identify comorbidities in the study population [23].
2. The Danish National Database of Reimbursed Prescriptions (DNDRP), used to identify prescriptions redeemed for inhaled corticosteroids (ICSs) and other medications at Danish community and hospital-based outpatient pharmacies since 1995 [24].
3. Microbiological data from the Clinical Microbiology Departments in Eastern Denmark (Region Zealand and Capital Region), consisting of approximately 2.6 million inhabitants, used to identify patients with *H. influenzae*.
4. The Danish register of Causes of Death, which provided mortality data [25].

Each registry is well validated and widely used in epidemiological research in Denmark.

### 2.2. Study Population

**Figure 1:**
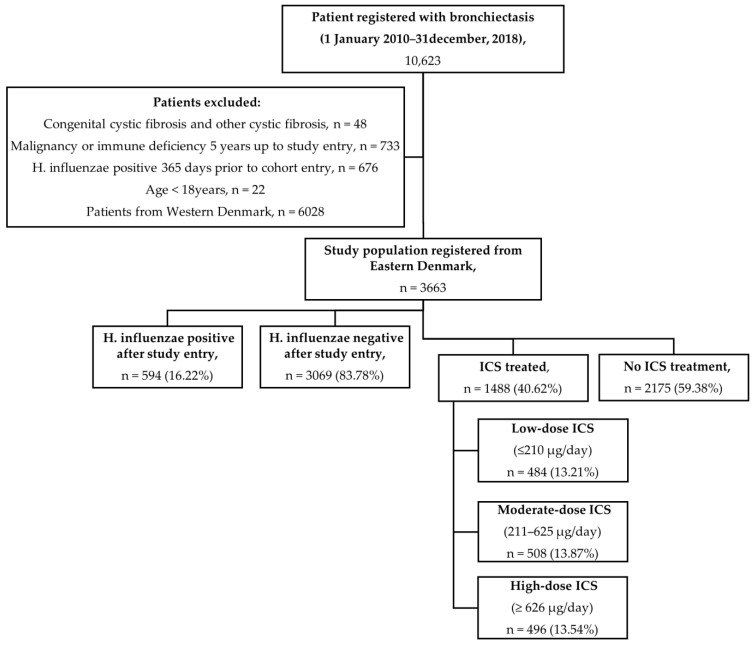
Study flow chart illustrating the patient selection criteria. A total of 3663 patients were included in this study, of whom 594 acquired *H. influenzae* during the study period. *H. influenzae*, *Haemophilus influenzae*; ICS, inhaled corticosteroid.

This observational cohort study includes all adults (≥18 years) with a diagnosis of BE who were managed at respiratory outpatient clinics in the Eastern part of Denmark from January 2010 to December 2018 (Figure 1). Cohort entry was defined as the date of the first outpatient visit to a respiratory department with the ICD-10 diagnosis code J47. Patients residing in Western Denmark were excluded because microbiological data were unavailable. *H. influenzae* was defined as any positive isolation from lower respiratory tract culture (i.e., sputum, tracheal secretion, bronchial secretion, and bronchial alveolar lavage) post-cohort entry. Patients with a *H. influenzae*-positive lower respiratory tract sample 12 months prior to cohort entry were excluded. We further excluded patients with cystic fibrosis, a history of malignant neoplasm or immunodeficiency 5 years before cohort entry, and/or redeemed prescription of disease-modifying anti-rheumatics drugs (Anatomical Therapeutic Chemical (ATC) codes: L04AX03, L01AA01, A07EC01, L04AD01, L04AA13, L04AX01, L04AA06, P01BA02) 12 months prior to cohort entry. These exclusions aimed to minimize potential confounding related to altered isolation risk. Supplementary Table S1 lists the ICD-10 codes used for the definition of comorbidities.

To assess the association between use of inhaled corticosteroids in patients with bronchiectasis and risk of *H. influenzae* in a dose-dependent manner, while adjusting for sex, age, COPD/asthma, and OCS dose, we aim to achieve a sample size of at least 3400 patients. This allows us to detect a significant hazard ratio of 1.35 between patients with non-use and high use (α = 0.05, power = 80%, expected event rate 0.25 in the high-dose group, and exposure ratio 0.22). The final cohort exceeded this threshold.

### 2.3. Exposure to ICSs

Exposure was defined as any redeemed prescription for ICS monotherapy or combination inhalers within the 12 months preceding cohort entry. All ICS doses were standardized to budesonide equivalent doses, as shown in Supplementary Table S2 [26]. Dose–response was assessed by dividing ICS exposure into tertiles: low (≤210 μg/day), moderate (211–625 μg/day), and high (≥626 μg/day), based on the accumulated dose in the year before cohort entry. Individuals with no ICS prescriptions served as the reference group.

### 2.4. Outcome and Follow-Up

The primary outcome was the first isolation of *H. influenzae* from lower respiratory tract samples (e.g., sputum, tracheal secretion, bronchial secretion, or bronchoalveolar lavage) post-cohort entry. Because microbiological data reflect the presence of the organism rather than clinical symptoms, the outcome represents isolation/colonization not confirmed infection.

Patients were followed for up to 5 years or until a positive *H. influenzae* lower airway tract sample, death, emigration, or the end of the observation period (31 December 2018).

### 2.5. Statistical Analysis

Continuous variables are presented as median values and interquartile ranges (IQRs). Categorical variables are reported as frequencies and proportions. A *p*-value ≤ 0.05 was considered statistically significant.

The association between *H. influenzae* and ICS use was estimated by using a cause-specific Cox proportional hazards regression model, accounting for death as a competing event. The model was adjusted for age (18–59 years vs. 60–69 years, 70–79 years, and >79 years of age), sex (female vs. male), concomitant COPD or asthma (not associated vs. associated), and accumulated dose of oral corticosteroids (no use vs. low dose and high dose) 12 months prior to cohort entry. Exposure to oral corticosteroids (OCSs) was divided into none, low (<625 mg), and high dose (≥625 mg), based on the median cumulative prednisone equivalent dose. Severity of bronchiectasis, lung function, symptoms, and smoking status were unavailable and are addressed in the limitations. Both the unadjusted results of univariate analyses for each covariate and the adjusted multivariate analyses, which included all covariates in the same model, were reported.

To evaluate the effect of treatment within specific subgroups, interaction analyses were conducted between ICS use and each of the following: age, sex, coexisting COPD/asthma, and OCS use.

To assess the robustness, we performed an inverse probability-of-treatment weighting (IPTW). Multinomial propensity scores were used to implement an IPTW-weighted cause-specific Cox proportional hazard model, based on the same variables as the adjusted primary analysis, to evaluate the robustness of findings.

Statistical analyses were performed using Statistical Analysis Software 9.4 (v.3.71 Enterprise Edition, SAS Institute, Mumbai, MSA, USA). Inverse probability-of-treatment weighting was performed in RStudio V.4.1.3 (R Foundation for Statistical Computing, Vienna, Austria) using the TWANG V.2.5 package.

### 2.6. Ethics

The authors were granted access to data in nationwide registers for this study in compliance with the current Danish laws (Data Protection Agency: P-2020-1223). According to Danish laws, ethics committee approval and informed consent are not required for register-based research.

## 3. Results

Between January 2010 and December 2018, 10,623 patients were identified with a diagnosis of bronchiectasis across respiratory outpatient clinics in Eastern Denmark. After applying the exclusion criteria, 3663 patients were included in the final analysis. This ensured adequate statistical power to detect clinically meaningful dose-dependent differences in the risk of *H. influenzae* isolation across ICS exposure groups by providing a cohort size larger than that required by our power assumptions. The mean age of the study population was 66 years, and 61% of the participants were female. Of these, 1488 patients (41%) had received ICSs within one year before cohort entry, including 484 (13.2%) at low dose, 508 (13.9%) at moderate dose, and 496 (13.5%) at high dose (Table 1).

Overall, 1920 patients (52.4%) had concomitant COPD or asthma, and 475 (13.0%) had both conditions. ICS use was more frequent in patients with COPD and asthma, whereas the prevalence of heart failure, myocardial infarction, and diabetes did not differ between exposure groups. Treatment with oral corticosteroids, long-acting β2-agonist (LABA), or long-acting muscarinic antagonist (LAMA) was more common among patients receiving high-dose ICS. Baseline characteristics are listed in Table 1.

During follow-up, in the entire cohort, 594 patients (16.2%) had a positive lower respiratory tract culture for *H. influenzae*. The median budesonide equivalent cumulative daily dose was higher among patients with *H. influenzae* isolation (575 µg) compared with that in those without detection (329 µg) (Table 2).

Budesonide and fluticasone propionate were the most frequently prescribed ICS agents (Supplementary Table S3).

During follow-up, 14,642 lower respiratory tract samples were obtained from 1953 patients. The most common bacterial pathogens were *H. influenzae* (n = 229, 22.0%), *Staphylococcus aureus* (n = 87, 8.4%), and *Moraxella catarrhalis* (n = 84, 8.1%).

### 3.1. Outcome Results

A total of 570 (15.56%) patients (distributed as no ICSs: 277; low ICSs: 63; moderate ICSs: 93; and high ICSs: 137) had first-time *H. influenzae* isolated from a lower respiratory tract culture, and 333 (9.09%) patients died within 5 years of cohort entry (Table 3). The median time to first-time *H. influenzae* isolation was 230 days (IQR: 32–637).

**Figure 2:**
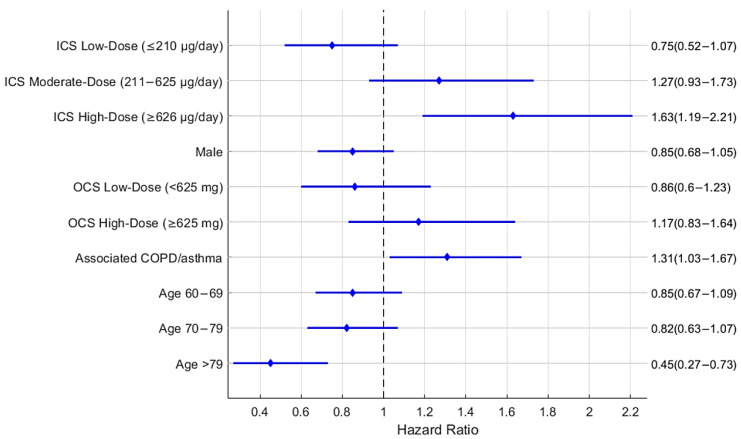
Forest plot of variables used in the Cox proportional hazard regression model of acquiring a lower respiratory tract sample positive for *H. influenzae*, showing adjusted hazard ratios with their respective 95% confidence intervals (CIs). The variables include accumulated daily ICS dose (low-dose ICSs (≤210 μg/day); moderate-dose ICSs (211–625 μg/day); high-dose ICSs (≥626 μg/day); reference group: no OCS use), accumulated OCS dose based on the median cumulative prednisone equivalent dose (low-dose OCSs (<625 mg) and high-dose OCSs (≥625 mg); reference group: no OCS use), age (60–69 years, 70–79 years, and >79 years; reference group: 18–59 years), sex (male; reference group: female), associated COPD/asthma (present; reference group: absent). The high-dose ICS category shows a statistically significant association. COPD, chronic obstructive pulmonary disease; ICSs, inhaled corticosteroids; OCSs, oral corticosteroids.

In an adjusted Cox regression analysis, high-dose ICS use was significantly associated with an increased risk of *H. influenzae* isolation compared with non-use (HR 1.63; 95% CI, 1.19–2.12; *p* < 0.005). No significant associations were observed for low-dose (HR 0.75; 95% CI, 0.52–1.07; *p* = 0.11) or moderate-dose ICSs (HR 1.27; 95% CI, 0.93–1.72; *p* = 0.12) (Figure 2). Unadjusted analyses demonstrated a dose–response trend, with higher hazard ratios in moderate-dose (HR 1.48; 95% CI, 1.12–1.97; *p* = 0.006) and high-dose ICS use (HR 1.97; 95% CI, 1.51–2.58; *p* < 0.0001) compared with non-use. Older age (>79 years) and concomitant COPD or asthma were also significant predictors, with coexisting COPD/asthma increasing the hazard by approximately 53% (HR 1.53; *p* < 0.0001). (Table 4).

**Figure 3:**
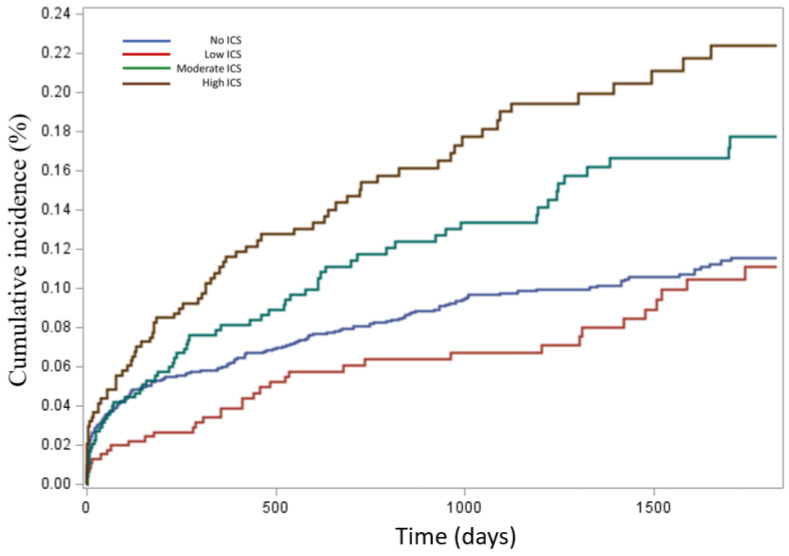
Cumulative incidence of positive lower respiratory tract sample for *H. influenzae* within 5 years of cohort entry according to exposure to accumulated doses of inhaled corticosteroids (ICSs) in the following four ICS groups: no ICS use (blue), low ICS dose (≤210 μg/day) (red), moderate ICS dose (211–625 μg/day) (green), and high ICS dose (≥626 μg/day) (brown).

Cumulative incidence curves illustrated that patients using high-dose ICSs had the greatest likelihood of *H. influenzae* detection over time, while low-dose users exhibited slightly reduced risk compared with non-users (Figure 3).

**Figure 4:**
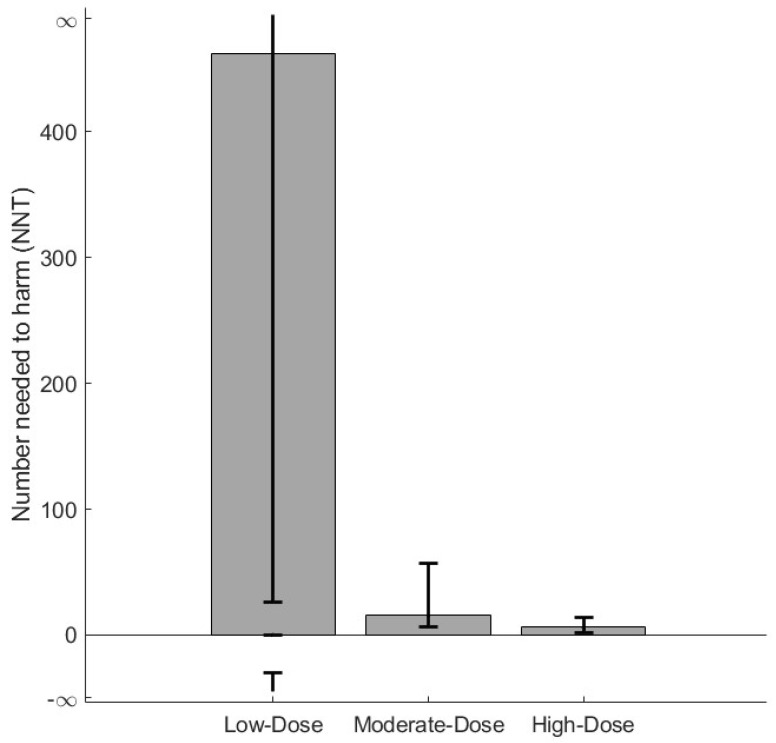
Number needed to harm (NNH) to acquire a lower respiratory tract sample positive for *H. influenzae* for low, moderate, and high ICS use. ICS exposure was determined based on the accumulated dose of ICS prescriptions reimbursed within 12 months prior to study entry. The accumulated dose was divided into tertiles: low-dose ICSs (≤210 μg/day); moderate-dose ICSs (211–625 μg/day); high-dose ICSs (≥626 μg/day); with non-ICS users serving as the reference group. The NNH for the three groups: low ICSs (NNH 462, 95% CI 26 (benefit) to ∞ to 29 (harm)), moderate ICSs (NNH 16, 95% CI 9.6–41.4), and high ICSs (NNH 6, 95% CI 4.6–8.0). *H. influenzae*, *Haemophilus influenzae*; ICSs, inhaled corticosteroids.

The number needed to harm (NNH) analysis estimated that 1 in 6 patients treated with high-dose ICS developed *H. influenzae*-positive cultures during follow-up (Figure 4, Supplementary Table S4).

### 3.2. Sensitivity Analyses

Interaction analysis revealed significant modification of risk by sex in the moderate-dose group (*p* = 0.01) and by age. No significant interactions were observed for coexisting COPD, asthma, or both, as well as for OCS use. Results for all interaction analyses are displayed in Table 5, Table 6, Table 7 and Table 8.

IPTW analysis confirmed the main findings: low-dose HR 0.87 (95% CI, 0.59–1.27; *p* = 0.46), moderate-dose HR 1.31 (95% CI, 0.91–1.87; *p* = 0.14), and high-dose HR 1.61 (95% CI, 1.02–2.54; *p* = 0.042) (Table 9).

## 4. Discussion

In this large multiregional cohort study of nearly 3000 patients with BE, we found that high-dose ICS use (≥626 μg/day) was independently associated with a 63% higher risk of *H. influenzae* isolation from lower respiratory tract cultures compared with non-use. No significant associations were observed for low- or moderate-dose ICSs. These findings were consistent across sensitivity analyses. Patients with concomitant COPD or asthma had a 1.5-fold higher risk of hazard.

We observed that individuals aged >79 years appeared to have a lower hazard of *H. influenzae* isolation. This likely reflects competing mortality and reduced sampling frequency rather than a biological protective effect. We adjusted using cause-specific Cox models to account for competing death, but residual bias may remain.

Our findings align with prior evidence from COPD populations, in which ICS use has been associated with an increased and dose-dependent risk of isolating *H. influenzae* [9]. In BE, data have been sparse. A recent study reported increased risk of Staphylococcus aureus isolation among high-dose ICS users (>1000 µg/day) [27], while Håkansson et al. observed associations with Pseudomonas aeruginosa colonization and mortality [11]. Conversely, Kapur et al. did not identify increased risk of Pseudomonas aeruginosa with short-term ICS therapy [13]. Collectively, these studies suggest a pathogen-specific effect of ICSs on airway colonization. In line with this, recent studies have reported an increased risk of *Stenotrophomonas maltophilia* isolation among high-dose ICS users (>800 µg/day) [28] as well as a dose-dependent association between ICS use and *Moraxella catarrhalis* isolation in patients with COPD [29].The frequent overlap between BE and COPD may complicate diagnosis and treatment decisions, as overlap prevalence between 8 and 69% [9,30]. This study observed that around 34% of the population has concomitant COPD. This overlap likely contributes to the relatively high use of ICSs observed in our cohort, including among patients without guideline-based indications. In our study, 9% of patients received ICSs despite lacking concomitant asthma or COPD.

An important consideration in interpreting our findings is that the microbiological outcome used in this study reflects *H. influenzae* isolation rather than confirmed clinical infection. In bronchiectasis, detection of *H. influenzae* in respiratory samples typically represents chronic airway colonization, which is biologically and clinically distinct from symptomatic infection. However, colonization contributes to airway inflammation and increases the risk of subsequent exacerbations.

Patients with BE frequently suffer from chronic colonization by potentially pathogenic microorganisms in the lower airways. The incidence of colonization with potential pathogenic microorganisms has been reported to be as high as 64% [31]. *H. influenzae* is the most frequent pathogen, found in 19–55% of patients [32]. Although it is less disruptive to the lower airway microbiota than *Pseudomonas aeruginosa*, *H. influenzae* can establish persistent infection through immune evasion when mucosal host defense mechanisms are impaired [17,33]. Corticosteroid-induced suppression of innate and adaptive immunity provides a biologically plausible explanation for the observed association between ICS use and *H. influenzae* isolation. ICSs may impair local host defenses by reducing macrophage phagocytic function, altering neutrophil chemotaxis, suppressing antimicrobial peptides, and weakening epithelial innate immune signaling via peptide transporters [14,34]. Thereby, ICSs contribute to impaired bacterial clearance and facilitate persistent colonization of non-encapsulated organisms such as *H. influenzae* [35]. Additionally, *H. influenzae* can persist through biofilm formation, and glucocorticoids have a direct influence on biofilm modulation by alternate gene expression [36]. This may explain the organism-specific susceptibility observed.

### 4.1. Strengths and Limitations

Strengths of this study include its large, well-characterized cohort, which exceeded the sample size required by our power analysis, providing confidence that the observed association for high-dose ICSs is unlikely to be due to limited statistical power. In addition, the cohort was a comprehensive registry linkage and complete regional microbiological data was used, allowing robust ascertainment of *H. influenzae* isolation across inpatient, outpatient, and primary care settings. Adjustment for multiple clinically relevant confounders and confirmation through sensitivity analyses further strengthen the validity of our findings.

Limitations include a lack of information on important clinical variables such as symptom severity, lung function (e.g., FEV_1_), smoking status, and body mass index. These factors reflect bronchiectasis severity and may influence both the decision to prescribe ICSs and the likelihood of obtaining respiratory cultures. Microbiological sampling was performed according to clinical indication, which may introduce selection bias. Actual adherence to ICS treatment could not be assessed, and misclassification of exposure is possible. Finally, due to the observational design, residual confounding cannot be excluded, and causality cannot be firmly established.

### 4.2. Clinical Implications

Our results highlight the potential risks associated with high-dose ICSs in BE patients, particularly those with coexisting COPD or asthma or both conditions. While ICSs may provide symptom relief in selected cases, clinicians should carefully weigh the benefits against the risk of isolation, especially in elderly patients or those at high risk of exacerbations.

## 5. Conclusions

In summary, high-dose ICS use in patients with BE was independently associated with increased risk of *H. influenzae* airway isolation, whereas low- and moderate-dose ICS use were not. Given the observational nature of the study, causality cannot be established, and residual confounding cannot be excluded. Nevertheless, these findings underscore the importance of cautious prescribing and careful risk–benefit assessment, particularly for patients with concomitant COPD or asthma or both conditions. Further prospective studies are required to clarify causality and underlying biological mechanisms to confirm these observations and explore potential pathogen-specific effects of ICSs in bronchiectasis.

## Figures and Tables

**Table 1:** Baseline characteristics of the study population at cohort entry.

	*H. influenzae* Negative n = 3069 (83.78%)	*H. influenzae* Positive n = 594 (16.22%)	Total (n = 3663)	No ICSs (n = 2175, 59.38%)	Low-Dose ICSs (≤210 μg/day), (n = 484, 13.21%)	Moderate-Dose ICSs (211–625 μg/day), (n = 508, 13.87%)	High-Dose ICSs (≥626 μg/day), (n = 496, 13.54%)
Sex, Male, n (%)	1213 39.52%	219 36.87%	1432 39.09%	895 41.15%	186 38.43%	185 36.42%	166 33.47%
**Age group, years, n%**							
Age, years, median (IQR)	66 (56–74)	64 (53–71)	66 (56–73)	66 (57–74)	64 (53–72)	65 (55–73)	65 (56–73)
<60	966 31.48%	227 38.22%	1193 32.57%	663 30.48%	188 38.84%	171 33.66%	171 34.47%
60–69	927 30.21%	184 30.98%	1111 30.33%	674 30.99%	140 28.93%	147 28.94%	150 30.24%
70–79	855 27.86%	148 24.92%	1003 27.38%	615 16.79%	113 23.35%	144 28.35%	131 26.41%
>79	321 10.46%	35 5.89%	356 9.72%	223 10.25%	43 8.88%	46 9.06%	44 8.87%
* Number of hospitalizations 12 months prior to cohort entry for all causes, n (%)							
1	2145 69.89%	390 65.66%	2535 71.47%	1545 71.03%	321 66.32%	343 67.52%	326 65.72%
≥2	821 26.75%	191 32.15%	1012 28.53%	559 25.7%	155 32.02%	144 28.35%	154 31.04%
**Comorbidities at cohort entry in the study population**							
COPD	985 32.10%	243 40.91%	1228 33.52%	507 23.31%	156 32.23%	258 50.79%	307 61.89%
Asthma	918 29.91%	249 41.92%	1167 31.86%	329 15.13%	185 32.22%	303 59.65%	350 70.56%
Hypertension	888 28.93%	165 27.78%	1053 28.75%	585 26.90%	135 27.8%	165 32.48%	168 33.87%
Atrial fibrillation	373 12.15%	91 15.32%	464 12.67%	254 11.68%	59 12.19%	75 14.76%	76 15.32%
Myocardial infarction	200 6.52%	32 5.38%	232 6.33%	144 6.62%	29 5.99%	28 5.51%	31 6.25%
Heart failure	270 8.80%	58 9.76%	328 8.95%	173 7.95%	39 8.05%	60 11.81%	56 11.29%
Renal failure	120 3.91%	31 5.21%	151 4.12%	86 3.95%	16 3.30%	23 4.53%	26 5.24%
Peripheral vascular disease	200 6.52%	34 5.72%	234 6.39%	139 6.39%	28 5.78%	30 5.90%	37 7.47%
Cerebrovascular disease	323 10.52%	67 11.28%	390 10.65%	239 10.99%	48 9.92%	54 10.63%	49 9.88%
Diabetes mellitus type 1	101 3.29%	19 3.20%	120 3.28%	68 3.13%	18 3.72%	17 3.35%	17 3.43%
Diabetes mellitus type 2	256 8.34%	56 9.43%	312 8.52%	178 8.18%	45 9.30%	44 8.66%	45 9.07%
Systemic connective tissue disease	221 7.20%	43 7.24%	264 7.21%	164 7.54%	27 5.58%	42 8.26%	31 6.25%
Depression	128 4.17%	28 4.71%	156 4.26%	98 4.50%	12 2.48%	25 4.92%	21 4.23%
Malignancy	365 11.89%	81 13.63%	446 12.18%	292 13.42%	54 11.16%	51 10.03%	49 9.88%
Immune deficiency	56 1.82%	26 4.38%	82 2.24%	43 1.98%	9 1.86%	14 2.76%	16 3.22%
** Death within study period	386 12.58%	69 11.62%	455 12.42%	239 10.99%	52 10.74%	68 13.38%	96 19.35%
**Use of medication 12 months prior to cohort entry**							
OCSs, accumulated dose, mg, median (IQR)	0 (0–500)	250 (0–500)	0 (0–500)	0 (0–250)	0 (0–500)	250 (0–750)	500 (250–1000)
No use, n (%)	2494 81.26%	441 74.24%	2935 80.13%	1955 89.88%	380 78.51%	325 63.98%	275 55.44%
OCS use, n (%)	575 18.74%	153 25.76%	728 19.87%	220 10.11%	104 21.49%	183 36.02%	221 44.55%
Low-dose OCSs (<625 mg), n (%)	304 9.91%	73 12.29%	377 10.29%	114 5.24%	70 14.46%	95 18.70%	98 19.75%
High-dose OCSs (≥625 mg), n (%)	271 8.84%	80 13.47%	351 9.58%	106 4.87%	34 7.02%	88 17.32%	123 34.80%
LABA, n (%)	205 6.68%	55 9.26%	260 7.10%	76 3.49%	30 6.20%	75 14.76%	79 15.93%
LAMA, n (%)	405 13.20%	113 19.02%	518 14.14%	104 4.78%	55 11.36%	155 30.51%	204 41.13%
Any use of antibiotics, n (%)	2145 69.89%	503 84.68%	2648 72.29%	1478 67.95%	363 75%	389 76.57%	418 84.27%

Data are presented as n (%) unless otherwise specified. * 12 months prior to study entry. ** Death within study period (1 January 2010–31 December 2018). COPD, chronic obstructive pulmonary disease; *H. influenzae*, *Haemophilus influenzae;* ICSs, inhaled corticosteroids; OCSs, oral corticosteroids; LABA, long-acting β2-agonist; LAMA, long-acting muscarinic antagonist; IQR, interquartile range.

**Table 2:** Overview of ICS use in the study population 12 months prior to cohort entry in the study population. Use of ICSs 12 months prior to cohort entry in 1488 (40.62%) patients with bronchiectasis. Patients with no ICS use (n = 2175, 59.38%) 12 months prior to cohort entry are not included in the table.

	*H. influenzae*-Positive ICS User (n = 305, 8.33%)	*H. influenzae*-Negative ICS User (n = 1183, 32.29%)	Total (n= 1488, 40.62%)
Accumulated equivalent ICS dose, mcg, median (IQR) *	575.34 (210.41–986.30)	328.77 (109.59–657.53)	335.34 (157.80–762.74)
ICS users in defined tertiles **, n (%)			
Low-dose ICSs (≤210 μg/day)	69 22.62%	414 35.00%	483 32.46%
Moderate-dose ICSs (211–625 μg/day)	95 31.15%	414 35.00%	509 34.21%
High-dose ICSs (≥626 μg/day)	141 46.23%	355 30.00%	496 33.33%
Number of individual users by ICS type ***, n (%)			
Budesonide	246 80.66%	1068 90.28%	1314 88.30%
Fluticasone propionate	130 42.62%	394 33.30%	524 35.21%
Fluticasone furoate	11 3.60%	44 3.72%	55 3.70%
Beclomethasone	20 6.56%	125 3.41%	145 9.75%
Mometasone	4 1.31%	13 10.57%	17 1.14%
Ciclesonide	5 1.64%	59 4.99%	64 4.30%
**Number of mono- or combinations users, n (%)**			
Mono	134 43.93%	627 53.00%	761 51.14%
2-drugs combination	263 86.23%	1001 84.61%	1264 84.95%
3-drugs combination	8 2.62%	31 2.62%	39 2.63%

Data are reported as n (%) or median (IQR = interquartile range), unless indicated otherwise. * Budesonide equivalent doses were calculated using the following ratio: beclomethasone 1:1, mometason1:1, ciclesonide 2:1, fluticasone propionate 2:1, fluticasone furoate 10:1. ** The accumulated budesonide equivalent ICS dose during the year preceding cohort entry was used as the basis for classification. The ICS equivalent dose was divided into three tertiles: low-dose ICSs (≤ 210 μg/day); moderate-dose ICSs (211–625 μg/day); high-dose ICSs (≥626 μg/day). *** Please note that individuals may have been prescribed more than one type of ICS. *H. influenzae*, *Haemophilus influenzae*; ICSs, inhaled corticosteroids; IQR, interquartile range.

**Table 3:** Outcomes within 5 years following cohort entry.

Outcomes	No ICSs	Low-Dose ICSs (≤210 μg/day)	Moderate-Dose ICSs (211–625 μg/day)	High-Dose ICSs (≥626 μg/day)	Total
First-time *Haemophilus influenzae* isolation, n (%)	277 7.56%	63 1.72%	93 2.54%	137 3.74	570 15.56%
Death, n (%)	188 5.13%	39 1.06%	51 1.39%	55 1.50	333 9.09%

ICSs: inhaled corticosteroids; the ICS equivalent dose was divided into 3 tertiles: low, moderate, and high, based on the ICS accumulated budesonide equivalent dose during the year preceding cohort entry.

**Table 4:** Cox proportional hazards regression results with both unadjusted and adjusted confounders.

	Unadjusted Hazard Ratio				Adjusted Hazard Ratio			
Parameter	Hazard Ratio	95%HR Confidence Interval		*p*-Value	Hazard Ratio	95%HR Confidence Interval		*p*-Value
No ICS treatment	Ref.				Ref.			
Low-dose ICSs (≤210 μg/day)	0.82	0.57	1.17	0.264	0.75	0.52	1.07	0.115
Moderate-dose ICSs (211–625 μg/day)	1.48	1.12	1.97	0.006	1.27	0.93	1.73	0.127
High-dose ICSs (≥626 μg/day)	1.97	1.51	2.58	<0.0001	1.63	1.19	2.21	<0.005
Female	Ref				Ref			
Male	0.83	0.67	1.03	0.096	0.85	0.68	1.05	0.132
No OCSs	Ref				Ref			
Low-dose OCSs: <625 mg	1.02	0.72	1.44	0.931	0.86	0.60	1.23	0.402
High-dose OCSs: ≥625 mg	1.46	1.06	2.01	0.022	1.17	0.83	1.64	0.376
No associated COPD or asthma or both	Ref				Ref			
Associated COPD or asthma or both	1.53	1.23	1.88	<0.0001	1.31	1.03	1.67	0.023
Age 18–59	Ref				Ref			
Age 60–69	0.85	0.66	1.09	0.190	0.85	0.67	1.09	0.210
Age 70–79	0.82	0.64	1.06	0.135	0.82	0.63	1.07	0.140
Age >79	0.45	0.28	0.74	<0.005	0.45	0.27	0.73	<0.005

Results from cause-specific Cox proportional hazards regression for 5 years follow-up after cohort entry for ICS users in tertiles divided doses adjusted with age, sex, concomitant COPD or asthma or both, and OCS used 1 year before cohort entry. Reference: age group: 18–59 years old; sex: female; ICS: not an ICS user; OCS: not an OCS user; concomitant COPD or asthma or both: not associated; ICSs: inhaled corticosteroids; OCSs: oral corticosteroids; COPD: chronic obstructive pulmonary disease.

**Table 5:** Interaction analysis for age.

Parameter	18–59 Years	60–69 Years	70–79 Years	>79 Years
No ICS treatment	Ref.	Ref.	Ref.	Ref.
Low-dose ICSs (≤210 μg/day)	Ref.	0.589	0.733	<0.005
Moderate-dose ICSs (211–625 μg/day)	Ref.	0.016	0.607	0.006
High-dose ICSs (≥626 μg/day)	Ref.	0.348	0.082	0.945

*p* values for interaction analysis for age. ICSs: inhaled corticosteroids; the ICS equivalent dose was divided into three tertiles: low, moderate, and high, based on the ICS accumulated budesonide equivalent dose 12 months before cohort entry.

**Table 6:** Interaction analysis for sex.

Parameter	Female	Male
No ICS treatment	Ref.	Ref.
Low-dose ICSs (≤210 μg/day)	Ref.	0.388
Moderate-dose ICSs (211–625 μg/day)	Ref.	0.010
High-dose ICSs (≥626 μg/day)	Ref.	0.680

*p* values for interaction analysis for sex. ICSs: inhaled corticosteroids; the ICS equivalent dose was divided into three tertiles: low, moderate, and high, based on the ICS accumulated budesonide equivalent dose 12 months before cohort entry.

**Table 7:** Interaction analysis for OCS dose.

Parameter	No OCS Treatment	Low-Dose OCSs <625 mg	High-Dose OCSs ≥625 mg
No ICS treatment	Ref.	Ref.	Ref.
Low-dose ICSs (≤210 μg/day)	Ref.	0.608	0.619
Moderate-dose ICSs (211–625 μg/day)	Ref.	0.348	0.173
High-dose ICSs (≥626 μg/day)	Ref.	0.163	0.760

*p* values for interaction analysis for OCS dose. ICSs: inhaled corticosteroids; the ICS equivalent dose was divided into three tertiles: low, moderate, and high, based on the ICS accumulated budesonide equivalent dose 12 months before cohort entry.

**Table 8:** Interaction analysis for concomitant COPD or asthma or both.

Parameter	No Concomitant COPD or Asthma or Both	Concomitant COPD or Asthma or Both
No ICS treatment	Ref.	Ref.
Low-dose ICSs (≤210 μg/day)	Ref.	0.476
Moderate-dose ICSs (211–625 μg/day)	Ref.	0.710
High-dose ICSs (≥626 μg/day)	Ref.	0.957

*p* values for interaction analysis for concomitant asthma and COPD. ICSs: inhaled corticosteroids; the ICS equivalent dose was divided into three tertiles: low, moderate, and high, based on the ICS accumulated budesonide equivalent dose 12 months before cohort entry. OCSs: oral corticosteroids; based on the median cumulative prednisone equivalent accumulated OCS dose.

**Table 9:** The IPTW (inverse probability-of-treatment-weighted sensitivity) hazard ratio.

Parameter	Hazard Ratio	95%HR Confidence Interval		*p*-Value
No ICS treatment	Ref.			
Low-dose ICSs (≤210 μg/day)	0.87	0.59	1.27	0.46
Moderate-dose ICSs (211–625 μg/day)	1.31	0.91	1.87	0.14
High-dose ICSs (≥626 μg/day)	1.61	1.02	2.54	0.042

ICSs: inhaled corticosteroids; the ICS equivalent dose was divided into three tertiles: low, moderate, and high, based on the ICS accumulated budesonide equivalent dose 12 months before cohort entry.

## Data Availability

Restrictions apply to the availability of these data. Data were obtained from the Danish National Health Authority and are available at https://sundhedsdatastyrelsen.dk/da/forskerservice/ansog-om-data (accessed on 21 May 2025) with the permission of the Danish National Health Authority.

## Supplementary Materials

The following supporting information can be downloaded at https://www.mdpi.com/article/10.3390/jcm14238557/s1: Table S1. International classification of Diseases 10th revision (ICD-10) used for the definition of comorbidities in the study population. Table S2: Equipotent doses of the different inhaled corticosteroid drugs analyzed. Table S3. Overview of ICS tertiles by type. Table S4. Number needed to harm (NNH) in different ICS groups compared to the control group.

## Abbreviations

The following abbreviations are used in this manuscript:

ATC-code	Anatomical therapeutic chemical code
BE	Non-cystic fibrosis bronchiectasis
CI	Confidence interval
COPD	Chronic obstructive pulmonary disease
CRS	Danish Civil Registration System
DNDRP	Danish Database of Reimbursed Prescriptions
DNPR	Danish National Patient Register
*H. influenzae*	*Haemophilus influenzae*
HR	Hazard ratio
ICD-10	International classifications of disease 10th revision
ICSs	Inhaled corticosteroids
IQR	Interquartile range
IPTW	Inverse probability-of-treatment weighting
LABA	Long-acting β2-agonist
LAMA	Long-acting muscarinic antagonist
OCSs	Oral corticosteroids

## References

[1] Barker A.F. (2002). Bronchiectasis. N. Engl. J. Med..

[2] Aliberti S., Goeminne P.C., O’Donnell A.E., Aksamit T.R., Al-Jahdali H., Barker A.F., Blasi F., Boersma W.G., Crichton M.L., De Soyza A. (2022). Criteria and definitions for the radiological and clinical diagnosis of bronchiectasis in adults for use in clinical trials: International consensus recommendations. Lancet Respir. Med..

[3] Choi H., McShane P.J., Aliberti S., Chalmers J.D. (2024). Bronchiectasis management in adults: State of the art and future directions. Eur. Respir. J..

[4] Keistinen T., Saynajakangas O., Tuuponen T., Kivela S. (1997). Bronchiectasis: An orphan disease with a poorly-understood prognosis. Eur. Respir. J..

[5] Wang L., Wang J., Zhao G., Li J. (2024). Prevalence of bronchiectasis in adults: A meta-analysis. BMC Public Health.

[6] Imam J.S., Duarte A.G. (2020). Non-CF bronchiectasis: Orphan disease no longer. Respir. Med..

[7] Barker A.F., Karamooz E. (2025). Non-Cystic Fibrosis Bronchiectasis in Adults: A Review. JAMA.

[8] Crimi C., Ferri S., Campisi R., Crimi N. (2020). The Link between Asthma and Bronchiectasis: State of the Art. Respiration.

[9] Martinez-Garcia M.A., Miravitlles M. (2017). Bronchiectasis in COPD patients: More than a comorbidity?. Int. J. Chronic Obstr. Pulm. Dis..

[10] Cole P.J. (1986). Inflammation: A two-edged sword--the model of bronchiectasis. Eur. J. Respir. Dis. Suppl..

[11] Håkansson K.E.J., Fjaellegaard K., Browatzki A., Dönmez Sin M., Ulrik C.S. (2021). Inhaled Corticosteroid Therapy in Bronchiectasis is Associated with All-Cause Mortality: A Prospective Cohort Study. Int. J. Chronic Obstr. Pulm. Dis..

[12] Chalmers J.D., Mall M.A., McShane P.J., Nielsen K.G., Shteinberg M., Sullivan S.D., Chotirmall S.H. (2024). A systematic literature review of the clinical and socioeconomic burden of bronchiectasis. Eur. Respir. Rev..

[13] Kapur N., Petsky H.L., Bell S., Kolbe J., Chang A.B. (2018). Inhaled corticosteroids for bronchiectasis. Cochrane Database Syst. Rev..

[14] Martínez-García M.Á., Oscullo G., García-Ortega A., Matera M.G., Rogliani P., Cazzola M. (2022). Inhaled Corticosteroids in Adults with Non-cystic Fibrosis Bronchiectasis: From Bench to Bedside. A Narrative Review. Drugs.

[15] Polverino E., Goeminne P.C., McDonnell M.J., Aliberti S., Marshall S.E., Loebinger M.R., Murris M., Cantón R., Torres A., Dimakou K. (2017). European Respiratory Society guidelines for the management of adult bronchiectasis. Eur. Respir. J..

[16] Chalmers J.D., Haworth C.S., Flume P., Long M.B., Burgel P.R., Dimakou K., Blasi F., Herrero-Cortina B., Dhar R., Chotirmall S.H. (2025). European Respiratory Society Clinical Practice Guideline for the Management of Adult Bronchiectasis. Eur. Respir. J..

[17] Roland N.J., Bhalla R.K., Earis J. (2004). The local side effects of inhaled corticosteroids: Current understanding and review of the literature. Chest.

[18] Wagner C., Goldmann T., Rohmann K., Rupp J., Marwitz S., Rotta Detto Loria J., Limmer S., Zabel P., Dalhoff K., Drömann D. (2015). Budesonide Inhibits Intracellular Infection with Non-Typeable Haemophilus influenzae Despite Its Anti-Inflammatory Effects in Respiratory Cells and Human Lung Tissue: A Role for p38 MAP Kinase. Respiration.

[19] Mohsin R.U., Heerfordt C.K., Eklöf J., Sivapalan P., Saeed M.I., Ingebrigtsen T.S., Nielsen S.D., Harboe Z.B., Iversen K.K., Bangsborg J. (2022). Use of Inhaled Corticosteroids and Risk of Acquiring Haemophilus influenzae in Patients with Chronic Obstructive Pulmonary Disease. J. Clin. Med..

[20] Chatziparasidis G., Kantar A., Grimwood K. (2023). Pathogenesis of nontypeable Haemophilus influenzae infections in chronic suppurative lung disease. Pediatr. Pulmonol..

[21] De Angelis A., Marchello M., Tramontano A., Cicchetti M., Nigro M., Simonetta E., Scarano P., Polelli V., Artuso V.A., Aliberti S. (2025). Haemophilus influenzae in bronchiectasis. Eur. Respir. Rev..

[22] Yang S.-H., Song M.J., Kim Y.W., Kwon B.S., Lim S.Y., Lee Y.-J., Park J.S., Cho Y.-J., Lee J.H., Lee C.-T. (2024). Understanding the effects of Haemophilus influenzae colonization on bronchiectasis: A retrospective cohort study. BMC Pulm. Med..

[23] Lynge E., Sandegaard J.L., Rebolj M. (2011). The Danish National Patient Register. Scand. J. Public Health.

[24] Schmidt M., Schmidt S.A.J., Adelborg K., Sundbøll J., Laugesen K., Ehrenstein V., Sørensen H.T. (2019). The Danish health care system and epidemiological research: From health care contacts to database records. Clin. Epidemiol..

[25] Helweg-Larsen K. (2011). The Danish Register of Causes of Death. Scand. J. Public Health.

[26] Ronn C., Sivapalan P., Eklof J., Kamstrup P., Biering-Sorensen T., Bonnesen B., Harboe Z.B., Browatzki A., Kjærgaard J.L., Meyer C.N. (2023). Hospitalization for chronic obstructive pulmonary disease and pneumonia: Association with the dose of inhaled corticosteroids. A nation-wide cohort study of 52 100 outpatients. Clin. Microbiol. Infect..

[27] Filipsen A.A., Frost K.H., Eklöf J., Tønnesen L.L., Vognsen A.K., Boel J.B., Pinholt M., Andersen C.Ø., Dessau R.B.C., Biering-Sørensen T. (2025). Inhaled Corticosteroids and Risk of Staphylococcus aureus Isolation in Bronchiectasis: A Register-Based Cohort Study. J. Clin. Med..

[28] Rønn C., Kamstrup P., Heerfordt C.K., Sivapalan P., Eklöf J., Boel J.B., Ostergaard C., Dessau R.B., Moberg M., Janner J. (2024). Inhaled corticosteroids and Stenotrophomonas maltophilia in outpatients with chronic obstructive pulmonary disease: A retrospective cohort study. BMJ Open Respir. Res..

[29] Johnsen R.H., Heerfordt C.K., Boel J.B., Dessau R.B., Ostergaard C., Sivapalan P., Eklöf J., Jensen J.-U.S. (2023). Inhaled corticosteroids and risk of lower respiratory tract infection with Moraxella catarrhalis in patients with chronic obstructive pulmonary disease. BMJ Open Respir. Res..

[30] Celli B.R., Fabbri L.M., Aaron S.D., Agusti A., Brook R.D., Criner G.J., Franssen F.M.E., Humbert M., Hurst J.R., de Oca M.M. (2023). Differential Diagnosis of Suspected Chronic Obstructive Pulmonary Disease Exacerbations in the Acute Care Setting: Best Practice. Am. J. Respir. Crit. Care Med..

[31] Angrill J., Agustí C., de Celis R., Rañó A., Gonzalez J., Solé T., Xaubet A., Rodriguez-Roisin R., Torres A. (2002). Bacterial colonisation in patients with bronchiectasis: Microbiological pattern and risk factors. Thorax.

[32] Borekci S., Halis A.N., Aygun G., Musellim B. (2016). Bacterial colonization and associated factors in patients with bronchiectasis. Ann. Thorac. Med..

[33] Finney L.J., Ritchie A., Pollard E., Johnston S.L., Mallia P. (2014). Lower airway colonization and inflammatory response in COPD: A focus on Haemophilus influenzae. Int. J. Chronic Obstr. Pulm. Dis..

[34] Schleimer R.P. (2004). Glucocorticoids suppress inflammation but spare innate immune responses in airway epithelium. Proc. Am. Thorac. Soc..

[35] Singanayagam A., Glanville N., Cuthbertson L., Bartlett N.W., Finney L.J., Turek E., Bakhsoliani E., Calderazzo M.A., Trujillo-Torralbo M.-B., Footitt J. (2019). Inhaled corticosteroid suppression of cathelicidin drives dysbiosis and bacterial infection in chronic obstructive pulmonary disease. Sci. Transl. Med..

[36] Xiao J., Su L., Huang S., Liu L., Ali K., Chen Z. (2023). Epidemic Trends and Biofilm Formation Mechanisms of Haemophilus influenzae: Insights into Clinical Implications and Prevention Strategies. Infect Drug Resist..

