# Supplementary material for: Inhaled Corticosteroid Use and Risk of Haemophilus influenzae Isolation in Patients with Bronchiectasis: A Retrospective Cohort Study
Source: J Clin Med. 2025 Dec 2;14(23):8557. doi: 10.3390/jcm14238557 (PMC12692804; doi:10.3390/jcm14238557)
Supplement: Supplementary file 1 [file jcm-14-08557-s001.zip › jcm-3963923-supplementary.pdf]

Supplementary Materials

Content

Supplementary Table S1. International classification of Diseases 10th revision (ICD-10) used for the definition of comorbidities in the study population 2

Supplementary Table S2. Equipotent doses of the different inhaled corticosteroid drugs analyzed...... 3

Supplementary Table S3. Overview of ICS tertiles by type........ 4

Supplementary Table S4. Number needed to harm (NNH) in different ICS group compared to no control group 5

Supplementary Table S1. International classification of Diseases 10th revision (ICD-10) used for the definition of comorbidities in the study population.

**Comorbidity**	**ICD-10 code**
COPD	J44
Asthma	J45
Hypertension	I10-115
Atrial fibrillation	I48
Myocardial infarction	I21-I23
Heart failure	I110, I130, I132, I42, I50, J81
Renal failure	I120, I131, I132; E102, E112, E132, E142 N02-N08, N11, N14, N158; N159, N160, N162- N164, N165 N168, N18, N19, N26, Z992
Peripheral vascular disease	I700-I174, I177, I179
Cerebrovascular disease	I60-I64, G45, G46
Diabetes mellitus type 1	E10
Diabetes mellitus type 2	E11-14
Systemic connective tissue disease	M05-09, M30-36, D86
Depression	F32-34
Malignancy	C00- D49
Immune deficiency	D80-84, D89
Bronchiectasis	J47

COPD: Chronic obstructive pulmonary disease

Supplementary Table S2. Equipotent doses of the different inhaled corticosteroid drugs analyzed

Drug	Dose (microgram; μg)
Budesonide	100
Mometasone	100
Beclomethasone	100
Beclomethasone HFA	50
Fluticasone propionate	50
Fluticasone furoate	10
Ciclesonide	40

HFA, Hydrofluoroalkane-134a.

Supplementary Table S3. Overview of ICS tertiles by type.
Based on ICS accumulated budesonide equivalent dose 1 year prior to cohort entry, the ICS equivalent dose was divided into 3 tertiles: low dose ICS (⩽ 210 μ g/day); moderate dose ICS (211-625 μ g/day); high dose ICS (⩾ 626 μ g/day).

	**Low dose ICS** (≤ 210 μg/day), (n= 484, 13.21%)	**Moderate dose ICS** (211-625 μg/day), (n= 508, 13.87%)	**High dose ICS** (≥ 626 μg/day), (n= 496, 13.54%)
Accumulated daily budesonide equivalent dose*(g), median (IQR)	105.20 (54.79-157.80)	328.77 (263.01-479.45)	986.30 (762.74 – 1502.47)
**Number of individual users by ICS type***, n (%)**			
Budesonide	379 (78.30%)	377 (74.21%)	205 (41.33%)
Fluticasone propionate	79 (16.32%)	98 (19.29%)	249 (50.20%)
Fluticasone furoate	5 (1.03%)	5 (0.98%)	12 (2.42%)
Beclomethasone	21 (4.34%)	20 (3.94%)	11 (2.22%)
Mometasone	1 (0.21%)	4 (0.79%)	9 (1.81%)
Ciclesonide	3 (0.62%)	10 (1.97%)	22 (4.43%)
**Number of mono or combinations user, n** (%)			
Mono	182 (37.60%)	183 (36.02%)	214 (43.14%)
2-stoffer	301 (62.19%)	325 (63.98%)	282 (56.85%)
3-stoffer	1 (0.21%)	0 (0%)	0 (0%)

Data are reported as n (%) or median (IQR = interquartile range), unless indicated otherwise.
* Budesonide equivalent doses were calculated using the following ratio: beclomethasone 1:1, Mometason1:1, Ciclesonide 2:1, Fluticasone propionate 2:1, Fluticasone furoate 10:1.
ICS, Inhaled corticosteroids; IQR, Interquartile range

Supplementary Table S4. Number needed to harm (NNH) in different ICS group compared to no control group

	No ICS treatment	Low dose ICS (≤ 210µg/day)	Moderate dose ICS (211-625µg/day)	High dose ICS (≥ 626µg/day)
**Attributable Risk %**	Ref.	0.22	6.41	17.13
**AR 95% CI**	Ref.	-3.36 % to 3.80%	2.42% to 10.40%	12.55% to 21.70%
**NNH**	Ref.	462	16	6
**NNH 95% CI**	Ref.	26 (benefit) to ∞ to 29 (harm)	9.6- 41.4	4.6-8.0

ICS: inhaled corticosteroids, the ICS equivalent dose was divided into 3 tertiles: low, moderate and high, based on ICS accumulated budesonide equivalent dose 1 year prior to cohort entry.
AR, Attributable Risk; NNH, Number needed to harm; CI, Confidence interval
